# Impact of the time elapsed between ICU request and actual admission on mortality and length of stay

**DOI:** 10.1186/cc12425

**Published:** 2013-03-19

**Authors:** GM Filho, TA Silva, AR Santana, FB Soares, LJ Almeida, LG Godoy, TA Rodrigues, MO Maia, JA Neto, AP Amorim, EB Moura, FF Amorim

**Affiliations:** 1 Escola Superior de Ciências da Saúde, Brasília, Brazil; 2 Unidade de Terapia Intensiva Adulto do Hospital Santa Luzia, Brasilia, Brazil; 3 Liga Academica de Medicina Intensiva do Distrito Federal (LIGAMI), Brasilia, Brazil

## Introduction

Measures to ensure an appropriate early treatment for critically ill patients result in significant decreases in mortality [1,2]. This study aims to evaluate the impact of the time elapsed from request until admission to the ICU on mortality and ICU length of stay (LOS).

## Methods

A retrospective cohort study performed on patients in the ICU of Hospital Regional de Samambaia over a period of 4 years, from January 2008 to December 2011. The patients were allocated into two groups: patients who waited longer than 6 hours, long waiting period (LWP, *n *= 300); and patients whose waiting time was equal to or less than that period, short waiting period (SWP, *n *= 113).

## Results

In total, 413 patients were included, 300 of which belonged to the LWP group (65.4%). For the entire cohort, the mean APACHE II score was 19 ± 7, the mean age was 52 ± 22 years, and 211 patients were male (51.1%). The LWP group did not show difference in the APACHE II score (19 ± 7 vs. 18 ± 8, *P *= 0.13), but was older (55 ± 20 vs. 49 ± 23, *P *= 0.01). LWP also had a higher incidence of primary bloodstream infection (23.8% vs. 10.4%, *P *= 0.01) and catheter-associated urinary tract infection (10.2% vs. 1.9%, *P *= 0.01). LWP patients had higher mortality (37.8% vs. 25.9%, *P *= 0.02) and longer ICU LOS (21 ± 47 vs. 14 ± 18 days, *P *= 0.01). Relative risk for death in the LWP was 1.74 (95% CI: 1.11 to 2.72).

## Conclusion

Despite showing no significant differences on APACHE II scores from the SWP group, patients from the LWP group presented greater incidence of primary bloodstream infection, catheter-associated urinary tract infection, higher mortality outcomes and longer ICU LOS.
